# Management of Ectopic Pregnancy and the COVID-19 Pandemic

**DOI:** 10.1055/s-0041-1725937

**Published:** 2021-02-26

**Authors:** Rujittika Mungmunpuntipantip, Viroj Wiwanitkit

**Affiliations:** 1 Ajeenkya DY Patil University, Pune, Maharashtra, India

Dear Editor,

We would like to share ideas on the publication “Medical Treatment for Ectopic Pregnancy during the COVID-19 Pandemic.” Elito Júnior and Araujo Júnior 1 mentioned that the “ *clinical treatment of ectopic pregnancy by MTX or expectant management is an alternative during the COVID-19 pandemic. An early diagnosis and appropriate selection of treatment options are critical for the success of the treatment* .” 1 In fact, using methotrexate (MTX) as alternative treatment for ectopic pregnancy during COVID-19 is a useful approach, and can reduce risk as well as decrease the workload at the hospital. The patient selection is very important. However, it should be noted that MTX might cause renal impairment, 2 and COVID-19 has a trend to develop renal impairment due to the immunopathological process of infection. 3 Close monitoring of the renal function is needed in MTX therapy, and the adjustment of the dosage based on renal function is important. 4

Author's response

## Reply to “Management of Ectopic Pregnancy and the COVID-19 Pandemic”

Júlio Elito Júnior, ^1^ 0000-0003-1514-5504 Edward Araújo Júnior ^1^ 0000-0002-6145-2532

^1^ Department of Obstetrics, Escola Paulista de Medicina, Universidade Federal de São Paulo, São Paulo, SP, Brazil

Address for correspondence: Edward Araújo Júnior, Departmento de Obstetrícia, Escola Paulista de Medicina, Universidade Federal de São Paulo, Rua Botucatu 740, Vila Clementino, São Paulo, SP, 04023-062, Brazil (e-mail: araujojred@terra.com.br).

Dear Editor,

Thank you for your comments. The most important message of this letter was the correct selection of patients for the medical treatment with methotrexate (MTX). We mentioned in the third paragraph that one of the exclusion criteria was renal dysfunction. Therefore, before the treatment, blood samples of every patient were collected for some exams, and one of them was creatinine. High levels of creatine were an exclusion criterion for the MTX treatment. On the other hand, patients with normal levels of creatinine can be submitted to the medical treatment. For tubal pregnancies, we recommend a single dose of MTX (50 mg/m ^2^ ). The risk of renal impairment related to this dose is very rare. 1 However, close monitoring of the renal function is necessary in cases of non-tubal pregnancies submitted to the protocol of multiple doses of MTX.

Conflict of Interests

The authors have no conflict of interests to declare.
